# Non-invasive biomarkers of perioperative stress in ophthalmic surgeons: Heart rate variability, cortisol and salivary copeptin in a feasibility study

**DOI:** 10.1371/journal.pone.0354245

**Published:** 2026-07-23

**Authors:** Rubén Cabrera Beyrouti, Marina López-García, María Eugenia Torregrosa Quesada, Jorge Francés Ferre, Ezequiel Campos-Mollo, Rocío Alfayate Guerra, Adolfo Aracil-Marco

**Affiliations:** 1 Servicio de Oftalmología, Hospital Universitario Vega Baja, Alicante, Spain; 2 Servicio de Laboratorio de Análisis Clínicos, Hospital General Universitario Dr. Balmis, Alicante, Spain; 3 Instituto de Investigación Biomédica y Sanitaria de Alicante (ISABIAL), Instituto de Salud Carlos III, Alicante, Spain; 4 Department of Ophthalmology, Hospital Virgen de los Lirios, Foundation for the Promotion of Health and Biomedical Research of the Valencian Community (FISABIO), Alicante, Spain; 5 Instituto Universitario de Investigación “Centro de Investigación del Deporte”, Universidad Miguel Hernández de Elche, Elche, Spain; University of Pisa, ITALY

## Abstract

Surgeons are exposed to acute perioperative stress that may compromise performance and patient safety. Objective, non-invasive biomarkers are essential to quantify and manage this stress. This prospective, quasi-experimental feasibility study aimed to describe the acute dynamics of heart rate (HR), heart rate variability (HRV), salivary cortisol, and salivary copeptin in ophthalmic surgeons during routine cataract surgery, while assessing the feasibility of measuring copeptin in human saliva for the first time. Fifteen ophthalmologists from three public hospitals were assessed on a resting day (baseline) and on a surgical day involving routine cataract procedures. HR and HRV (SDNN, RMSSD, pNN50, LF/HF) were recorded with a Polar H9 chest strap and a validated mobile application, and saliva samples were collected for cortisol and copeptin (a neurohormone released by the posterior pituitary gland) at four perioperative time points (arrival at hospital, pre-scrub, immediately post-surgery, and 30 min post-surgery). Repeated-measures analyses and correlation tests were performed. At rest, participants showed a mean HR 72.5 ± 15 beats·min ⁻ ¹ and salivary cortisol 0.41 ± 0.29 µg·dl ⁻ ¹, and detectable salivary copeptin levels (2.3 ± 0.7 pmol·l ⁻ ¹). On the surgical day, HR (66.6 ± 8.8 beats·min ⁻ ¹) and cortisol (0.21 ± 0.10 µg·dl ⁻ ¹) decreased significantly at 30 min post-surgery compared with pre-intervention values (74.0 ± 11.3 beats·min ⁻ ¹ and 0.47 ± 0.30 µg·dl ⁻ ¹, respectively), consistent with stress relief, while HRV indices displayed inverse patterns. Although a slight elevation in copeptin levels was observed during the surgical day (from 2.07 ± 0.42 at baseline to 2.50 ± 0.67 2.3 ± 0.7 pmol·l ⁻ ¹ immediately before surgery), this change did not reach statistical significance. In conclusion, the performance of routine cataract surgery triggers specific neuroendocrine responses in ophthalmologists. These findings support the use of multi-modal biomarkers to monitor perioperative strain and its potential impact on surgical performance.

## Introduction

Given the inherent demands of their profession, healthcare workers are subjected to a wide range of stressors. Psychological stress is of particular concern to medical staff members performing surgical duties since it can impact surgical performance and patient safety [1,2], and may also have biological consequences [3]. Therefore, there is a growing need for non-invasive on-site systems that can help to measure acute stress in this specific population, particularly in relation to surgery.

While heart rate (HR) is a measurement of the mean number of heart beats per unit of time, heart rate variability (HRV) is based in the measurement of the differences in the interbeat intervals [4]. HRV has emerged as a promising tool for assessing stress among healthcare workers [5]. The wide availability of wearable technology -such as smartphones, non-invasive sensors, etc.-, has paved the way to estimate the effect of real-life stressors on HRV [6]. Numerous studies have demonstrated that elevated occupational stress is associated with reduced HRV, as shown by decreased parasympathetic activity [7,8]. HRV has been shown to respond consistently to stress in medical professionals during simulations, clinical procedures, and real emergencies [8]. Some HRV variables, such as the standard deviation of normal-to-normal intervals (SDNN), the root mean square of the successive differences between adjacent RR intervals (RMSSD, in ms), the percentage of normal-to-normal intervals exceeding 50 ms (pNN50), the low-frequency percentage (LF%) and the low-frequency to high-frequency ratio (LF/HF ratio) seem to be sensitive to acute, contextual stress in this population [8,9].

Additionally, the quantification of salivary biomarkers has also been used to measure stress in surgeons. Salivary cortisol is the most studied, since it has an excellent plasma-to-saliva ratio, but other molecules such as alpha-amylase, secretory IgA, and chromogranin A, which are mostly produced by the direct sympathetic activation of the salivary glands, have also been evaluated as surrogates of stress exposure in surgeons [1,5].

Copeptin, the C-terminal portion of the arginine vasopressin (AVP) precursor [9], has emerged as a novel biomarker of interest due to its high stability in blood. Physiologically, copeptin is synthesized in the hypothalamus and released from the posterior pituitary gland in equimolar amounts to AVP in response to osmotic or hemodynamic stress [10], showing long-lasting molecular stability at routine laboratory temperature conditions, as well as lower oscillations than the latter [11]. Since copeptin plasma concentrations increase in several acute health conditions and seems related to their outcomes, in the medical field, its significance lies in its ability to reflect individual stress levels in acute conditions, providing a more reliable measure than traditional markers in complex environments such as the operating room. This has been reinforced by several studies that have shown that copeptin plasma concentrations increase after exposure to psychosocial stress [12,13]. Like other molecular biomarkers, such as cortisol, the possibility of measuring copeptin in salivary fluid instead of blood, would be of great interest to increase its use in real-life settings. However, to the best of our knowledge, there are no previous reports in which copeptin has been measured in saliva in humans.

Therefore, the primary outcome of this work is to describe the acute dynamics of heart rate and cortisol in relation to a real surgical intervention -routine cataract surgery- in ophthalmologists, a population that, to the best of our knowledge, has not yet been studied in this context. As a secondary outcome, the feasibility of HRV parameters and copeptin measurement in saliva as stress biomarkers will be evaluated.

## Materials and methods

### Ethics

This study was authorized by the Ethics Committee of the Alicante General Hospital (CEIm reference: PI2022−049). The tenets of the Declaration of Helsinki were followed. The participants signed an informed consent before their enrolment and were able to withdraw from the study at any time. Anonymity of the participants was guaranteed. Recruitment took place between 01/02/2022 and 31/12/2023.

### Study design

This is a pilot, prospective, quasi-experimental study, with repeated measurements.

### Participants

After signing an informed consent, 15 ophthalmologists (6 women and 9 men), from three independent public hospitals serving an urban area, volunteered for the study. Ophthalmologists were specifically selected for this study because ophthalmic surgery represents a unique model of high-precision microsurgery. Unlike other surgical disciplines, it requires prolonged periods of microscopic focus and extremely fine motor control, which are associated with specific patterns of acute mental and physiological stress. By maintaining a homogeneous sample of ophthalmologists, we aimed to minimize confounding factors such as varying surgical ergonomics or physical exertion levels found in other specialties, thereby ensuring that the observed fluctuations are more directly attributable to the specific demands of microsurgical procedures.

The participants had a mean age of 40.8 ± 11.2 years and a body mass index of 23.8 ± 2.8 kg·m^-2^, and declared a mean surgical experience of 2195 hours (range: 28–8000 hours). Except for the expected differences in body mass index (21.5 ± 1.2 vs. 25.3 ± 2.6 kg·m^-2^, women vs. men, respectively; p = 0.007, t-test) no further differences were noticed at any variable between men and women at baseline.

### Intervention

**Fig 1:**
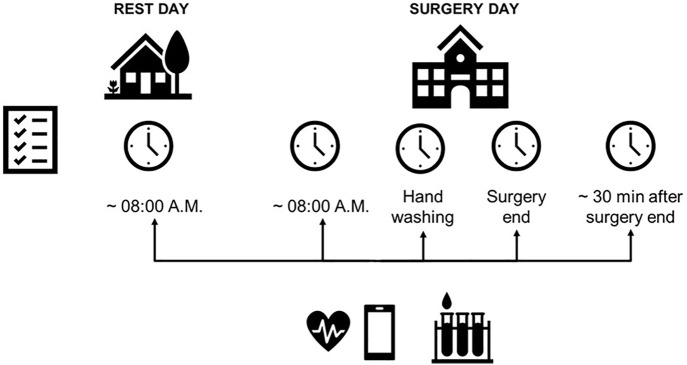
Diagrammatic representation of the experimental protocol. Before starting the study, the participants signed an informed consent and responded an ad hoc questionnaire. Heart rate, heart rate variability and saliva samples were obtained in two different days: a) during a rest day, at home; b) during a working day with surgical activity, in four occasions: at arrival at the hospital, immediately before and after surgery, and 30 min after the end of the surgery. Both days were separated by, at least, 48 h.

Participants were measured at two key time points. To establish a stable physiological baseline, samples were collected at 8:00 AM on a separate non-surgical rest day. These values were used as an independent reference to assess the participants’ basal state. The other four measurements were carried out sequentially along a morning in which the participants had surgical activity -routine cataract surgery- at the following time points: a) at arrival at the hospital early in the morning, approx. 30 min before the start of the surgery; b) immediately before the surgical hand-washing; c) immediately after the end of the surgery; and, d) approx. 30 min after the end of the surgery. On both days, all measurements were scheduled in the morning within a similar time window in order to reduce circadian variability in salivary cortisol. Fig 1 summarizes the study protocol.

#### Heart rate variability.

Heart rate variability was measured with a Polar® HR9 pulsimeter fixed to a chest band, and wireless connected to a mobile phone. Data were acquired with the validated application EliteHRV® [14].

For each measurement, the chest band was placed and the participant was seated in a relaxed position for five min. After that, 2 min of recording were obtained with the individual breathing spontaneously with their eyes open. The chest band was removed before surgery to avoid discomfort and reduce distraction of the surgeon. The automatic results provided by the app were extracted and annotated. From the variables provided by the app the heart rate (HR, in beats·min^-1^), SDNN (ms), RMSSD (ms), pNN50, and LF/HF ratio were included in the analyses since they seem to be sensitive to acute stress in medical professionals attending real or simulated emergencies [8]. Because of the constraints of the surgical workflow, we used ultra-short-term HRV recordings (< 5 min). This approach has practical advantages in real-world perioperative settings, although ultra-short HRV measurements may not fully reproduce longer-term autonomic dynamics.

#### Salivary collection and biochemical analysis.

During the 2 min of recording of HRV, the participants kept inside their oral cavity a swab (Salivette®, STARDET) under the anterior part of the tongue. The swabs were collected in the corresponding tubes and kept refrigerated at 4 ºC. Saliva samples were kept frozen at −20 ºC until analysis [15].

Cortisol concentration was determined by immunochemiluminescence (Cobas 411, Roche Diagnostics), and copeptin by immunofluorescence (Kryptor, Thermo Fisher), following the standard laboratory protocols of the Clinical Analysis Laboratory of Alicante General Hospital.

Water intake and hydration status were not standardized; participants followed their usual habits on both days.

### Statistical analysis

Unless otherwise indicated, continuous variables are shown as mean ± standard deviation, and categorical variables as number of cases (percentage). The normal distribution of the continuous data was verified by the Shapiro–Wilk or Kolmogorov-Smirnov tests and the equal variance was tested with the Levene test. The student’s t-test and the Pearson correlation test were used to look for differences and associations, respectively, between continuous variables with a normal distribution. The corresponding non-parametric test was used for non-normally distributed data. A one-way repeated-measures analysis of variance (ANOVA) with measurement order as a within-subjects factor was used to test for differences in the measured variables along the day of surgery. Bonferroni test was used for *post hoc* comparisons. Statistical comparisons between the rest-day baseline and the surgery-day baseline were performed using a Student’s t-test for paired samples. This measurement was intentionally excluded from the repeated measures ANOVA to focus the latter exclusively on the acute dynamic response to surgical stress throughout the operative day, thereby avoiding potential confounding effects from inter-day variability.

Differences were considered statistically significant at a critical level of p ≤ 0.05. All analyses were performed with the statistical package JASP (v.0.19.3.0) [16].

## Results and discussion

Mean values of all the analysed variables are summarized in the S1 Table.

### Resting day

On the resting day the participants exhibited a mean heart rate of 72.5 ± 15 beats·min^-1^, a SDNN of 54.7 ± 20.2 ms, a RMSSD of 34.1 ± 14.9 ms, a pNN50 of 13.2 ± 9.8 and a LF/HF ratio of 5.9 ± 6.8. Except for the expected difference in heart rate (82 ± 19 vs. 66.2 ± 6.9 beats·min^-1^, women vs. men, respectively; p = 0.031, t-test), no further significant differences were observed in any of these variables between men and women at baseline.

At baseline, salivary cortisol concentration was 0.41 ± 0.29 µg·dl^-1^, and the salivary copeptin concentration was 2.3 ± 0.7 pmol·l^-1^. No differences were noticed among these variables between men and women.

HRV variables showed a direct association between them, but were not associated with salivary cortisol or copeptin. Additionally, both hormonal salivary levels were not associated between them. Table 1 summarizes the correlation between the studied variables at baseline.

**Table 1:** Correlation matrix at the resting day.

Variable		HR		SDNN		RMSSD		pNN50	LF/HF ratio	Copeptin sal	Cortisol sal
**1. HR**	*R*	—									
	*p*	—									
**2. SDNN**	*R*	−0.523	*	—							
	*p*	0.045		—							
**3. RMSSD**	*R*	−0.497		0.891	***	—					
	*p*	0.060		< .001		—					
**4. pNN50**	*R*	−0.628	*	0.766	***	0.898	***	—			
	*p*	0.012		< .001		< .001		—			
**5. LF/HF ratio**	*R*	0.037		0.001		−0.253		−0.284	—		
	*p*	0.895		0.997		0.363		0.305	—		
**6. Copeptin sal**	*R*	0.281		−0.236		0.008		0.087	−0.346	—	
	*p*	0.330		0.417		0.979		0.766	0.226	—	
**7. Cortisol sal**	*R*	0.156		0.015		0.014		−0.074	−0.171	0.438	—
	*p*	0.580		0.956		0.960		0.792	0.543	0.117	—

* p < .05, ** p < .01, *** p < .001.

### Surgery day

In comparison with the resting day measurement, baseline SDNN increased significantly (68.3 ± 33.6 vs. 54.7 ± 20.1 ms, respectively; p = 0.023, paired t-test), and pNN50 showed a non-significant trend to increase (22 ± 18.2 vs 13.2 ± 9.8 ms, p = 0.088, paired t-test) in the first measurement taken on the surgery day. No other differences were noticed in the resting variables at the first measurement on the surgery day in comparison with the resting day

**Fig 2:**
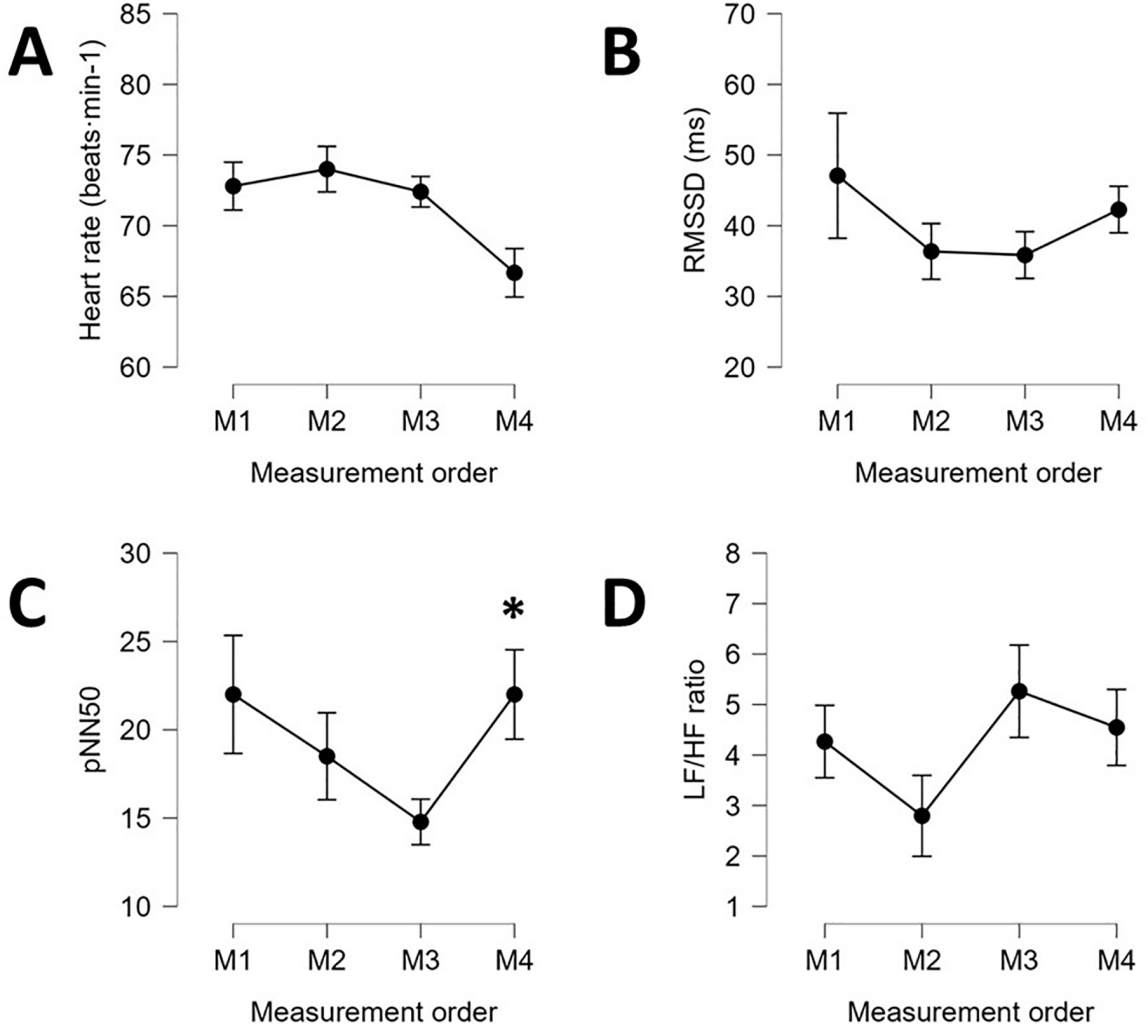
Response of the heart rate-related variables in relation to surgery. M1 to M4 indicate the measurement order. * p < 0.05, Bonferroni test, M4 vs M3. Data represent the mean ± standard error of the mean.

Fig 2 summarizes the evolution of the heart rate related variables taken during the consecutive measurements throughout the surgery day. Heart rate tended to decrease after the end of surgery (Fig 2A, p = 0.056, Holm-Sidak, M4 vs M2 and M3). RMSSD showed a trend to be decreased between the surgery start and end, and to recover afterwards (Fig 2B). Similarly, pNN50 showed a trend to decrease during the surgical procedure, and quickly returned to baseline values afterwards (Fig 2C). LF/HF showed a quick and transient decrease before the surgery, and returned to baseline immediately after the end (Fig 2D).

**Fig 3:**
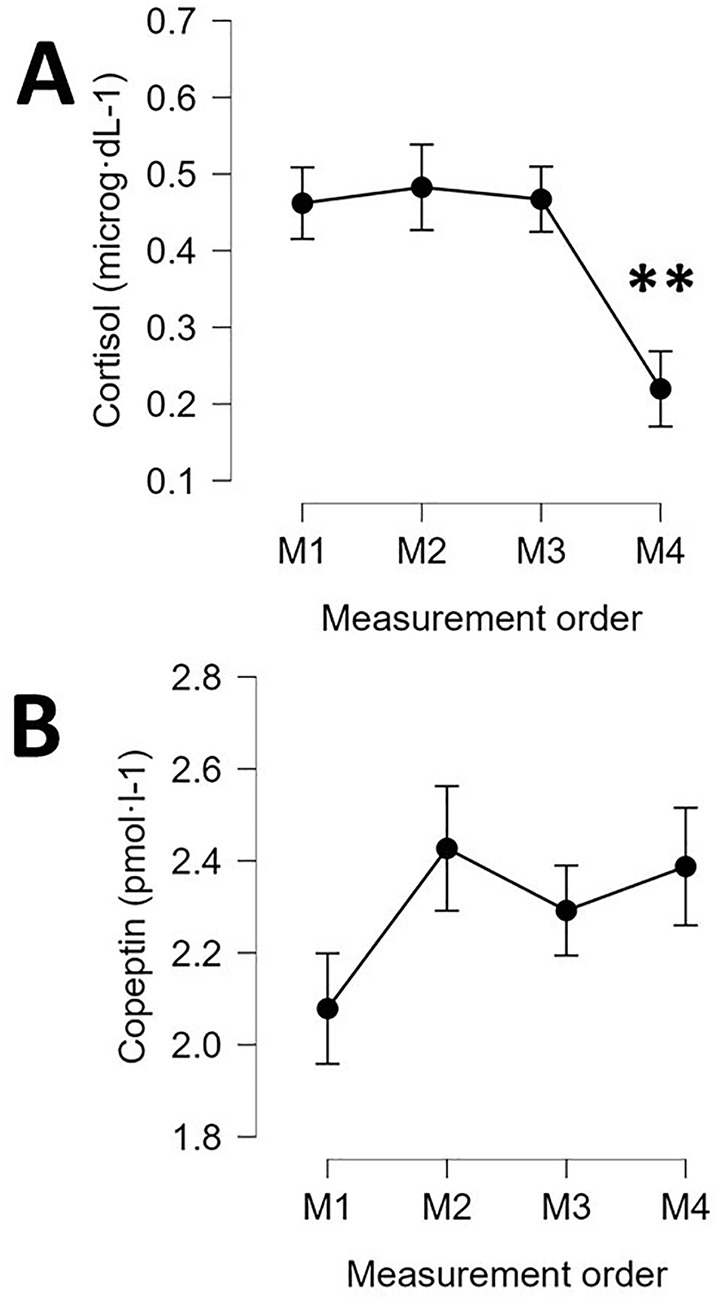
Cortisol and copeptin response in relation to surgery. M1 to M4 indicate the measurement order. * p < 0.05, Bonferroni test, M4 vs M1 and M3; ** p < 0.01, Bonferroni test, M4 vs. M1. Data represent the mean ± standard error of the mean.

Salivary cortisol paralleled the changes of heart rate (Fig 3A). Cortisol significantly decreased at M4 in comparison to the other measurements (p < 0.05 vs. M2 and M3; p < 0.01 vs M1, Bonferroni test). In contrast, salivary copeptin tended to increase at the beginning of the surgery, and kept elevated even after the end of the surgery (Fig 3B).

The mean values and standard deviation for each variable are summarized at S1 Table.

Overall, salivary copeptin negatively correlated with the parasympathetic activity variables RMSSD and pNN50 at surgery day (Table 2).

**Table 2:** Overall correlations during the surgery day.

Variable		HR		SDNN		RMSSD		pNN50		LF/HF ratio	Copeptin sal	Cortisol sal
1. HR	*R*	—										
	*p*	—										
2. SDNN	*R*	−0.164		—								
	*p*	0.211		—								
3. RMSSD	*R*	−0.134		0.865	***	—						
	*p*	0.306		< .001		—						
4. pNN50	*R*	−0.495	***	0.722	***	0.807	***	—				
	*p*	< .001		< .001		< .001		—				
5. LF/HF ratio	*R*	0.015		−0.010		−0.235		−0.262	*	—		
	*p*	0.908		0.938		0.071		0.045		—		
6. Copeptin sal	*R*	0.188		−0.157		−0.275	*	−0.325	*	0.120	—	
	*p*	0.154		0.236		0.035		0.013		0.365	—	
7. Cortisol sal	*R*	0.035		0.111		0.062		0.018		−0.004	−0.147	—
	*p*	0.794		0.403		0.643		0.892		0.973	0.266	—

*p < .05, ** p < .01, *** p < .001.

## Discussion

The present prospective, quasi-experimental feasibility study aimed to describe the dynamics of heart rate and salivary cortisol as non-invasive biomarkers in ophthalmologists in relation to their acute exposure to routine cataract surgery as an anxiogenic, real-world, work-specific situation. Repeated measurements were taken before and after surgery. Additionally, the feasibility of measurement of some HRV parameters as well as copeptin detection in saliva were also studied.

We found that after surgery heart rate and salivary cortisol decreased significantly, which can be interpreted as objective signs of stress alleviation. In relation to acute exposure to surgical stress, RMSSD, a surrogate of parasympathetic activity, showed a reverse pattern to heart rate, thus suggesting it as a potential HRV parameter sensitive to acute stress. The other HRV parameters (SDNN, pNN50) were less consistent in this regard. The explanation for this lack of consistency of all these HRV parameters is beyond of the scope of this study but may lie in the fact that we used an ultra-short-term measurement to avoid major interferences with the surgeries. Depending on the duration of HRV analysis, three main categories are usually considered: a) long-term (> 5 min), short-term (~ 5 min) and ultra-short term (< 5 min) measurements. Although all of them are based in the same metrics, ultra-short term HRV measurements may not reflect the same physiological changes that short or long-term measurements [17]. HRV in short-term recordings seems to be produced by four interdependent sources: (1) the complex interaction between the sympathetic and parasympathetic branches; (2) respiration-mediated increases and decreases in heart rate -respiratory sinus arrhythmia-; (3) the baroreceptor reflex that regulates blood pressure using negative feedback; and (4) rhythmic adjustments in blood vessel diameter. Short-term values correlate poorly with their long-term counterparts, and more basic research has been claimed to identify the major HRV generators in ultra-short recordings [17]. In our measurements neither breathing rhythm nor blood-pressure were controlled.

Regarding biochemical salivary responses to stress, salivary cortisol decreased quickly after the end of the cataract surgery, an observation that has been already reported in novice otorhinolaryngologists performing endoscopic sinus surgery [18]. Since either, acute and chronic stress seem to increase salivary cortisol [19] the elevated levels before and during the surgery may be interpreted as a tonic activity of the hypothalamic-pituitary-adrenal axis in the participating ophthalmologists. In this regard, cortisol seems to be associated with anxiety psychological traits [20], a variable that we haven’t studied. Additionally, the observed decrease of salivary cortisol after the surgery end may also reflect its well-known circadian rhythm, with peak levels early in the morning that decrease afterwards [21].

To the best of our knowledge salivary copeptin has only been previously measured in dogs [22]. Therefore, this would be the first study showing the feasibility of measurement of salivary copeptin in humans. In dogs, salivary copeptin was associated with the stress response to separation of the animals from their owners as an anxiogenic situation. Similarly, in our study salivary copeptin increased immediately before the surgery and kept elevated during the whole period of the study, in contrast to the dynamics of cortisol, that tended to decrease. The lack of correlation among biochemical stress markers has been previously reported in a similar context [18]. Since copeptin is released from the neurohypophysis, this unparallel behaviour may reflect a differential involvement of both parts of the pituitary gland during stress responses, a hypothesis that has been previously suggested [23]. Additionally, as recently reviewed, normal serum physiological copeptin levels demonstrate substantial variability [24]. Given that copeptin concentrations increase during water loss activities and that we didn’t control water intake during the measurement period, this could be another explanatory hypothesis for this observation. Moreover, since the mechanisms involved in copeptin elimination are still unclear [25], other hypotheses may not be ruled out currently. Finally, a correlational analysis of plasma-to-saliva ratio for copeptin needs to be performed in future studies. Therefore, while copeptin remains an interesting candidate for future research, its measurement in saliva as a stress biomarker marker needs further experimentation.

This feasibility study has some limitations that should be acknowledged. First, the sample size was small and restricted to ophthalmologists from three public hospitals, which limits the generalizability of the findings but is consistent with the exploratory nature of the study. Second, the use of ultra-short-term HRV recordings (2 minutes) was selected to balance the need for physiological data with the requirement of maintaining an unobtrusive environment for the surgeons. Although continuous recording throughout the surgical session could provide a more detailed temporal map of autonomic fluctuations, the potential for physical discomfort or distraction posed a risk to surgical ergonomics. Therefore, longer or continuous recordings could be considered in future studies to provide a more comprehensive view of the surgical stress profile. Third, although all measurements were scheduled in the morning, we did not strictly standardize the exact sampling times or participants’ sleep patterns, which may have influenced the circadian profile of cortisol. Fourth, water intake and hydration status were not controlled, which is an important consideration given the sensitivity of copeptin to osmotic changes.

Finally, we did not assess psychological traits such as baseline anxiety, which could modulate individual neuroendocrine activation. These factors should be addressed in larger, multicenter studies specifically designed to link surgeon stress responses with patient-related outcomes.

## Conclusions

In summary, RMSSD in ultra-short HRV measurements and salivary copeptin seem to be feasible future candidates to study the acute exposure to surgery-induced stress in ophthalmologists. Measuring them during the intervention, and during more prolonged time windows, would help to have a clearer picture of their potential future use to either detect the stress experienced by health workers and test the effectiveness of mitigation strategies that could be implemented in healthcare occupational settings.

## Supporting information

S1 Table Summary of the mean and standard deviation values for each measured variable. (DOCX)
