# Supplementary material for: Non-invasive biomarkers of perioperative stress in ophthalmic surgeons: Heart rate variability, cortisol and salivary copeptin in a feasibility study
Source: PLoS One. 2026 Jul 23;21(7):e0354245. doi: 10.1371/journal.pone.0354245 (PMC13395310; doi:10.1371/journal.pone.0354245)
Supplement: S1 Table — (DOCX) [file pone.0354245.s001.docx]

**Supplementary table 1. Summary of the mean and standard deviation values for each measured variable.**

Measurement 1 corresponds to the resting, non-surgery day and was take at 08:00 AM. Measurments 2-5 correspond to the sequential measurements taken during the surgery day, as described in the main text.

|  | **Measurement** | **Mean** | **Standard Deviation** |
| --- | --- | --- | --- |
| **Primary outcomes** |  |  |  |
| Heart rate (beats·min^-1^) | 1 | 72.533 | 15.023 |
|  | 2 | 72.800 | 12.451 |
|  | 3 | 74.000 | 11.326 |
|  | 4 | 72.400 | 9.455 |
|  | 5 | 66.667 | 8.861 |
| Salivary cortisol (µg·dl⁻¹) | 1 | 0.411 | 0.298 |
|  | 2 | 0.462 | 0.200 |
|  | 3 | 0.472 | 0.307 |
|  | 4 | 0.452 | 0.261 |
|  | 5 | 0.213 | 0.102 |
| **Secondary outcomes** |  |  |  |
| RMSSD (ms) | 1 | 34.146 | 14.971 |
|  | 2 | 47.078 | 38.571 |
|  | 3 | 36.361 | 16.852 |
|  | 4 | 35.848 | 17.724 |
|  | 5 | 42.275 | 19.865 |
| SDNN (ms) | 1 | 54.780 | 20.178 |
|  | 2 | 68.333 | 33.674 |
|  | 3 | 58.702 | 19.669 |
|  | 4 | 63.043 | 26.539 |
|  | 5 | 61.671 | 26.865 |
| pNN50 (%) | 1 | 13.200 | 9.858 |
|  | 2 | 22.000 | 18.191 |
|  | 3 | 17.400 | 16.690 |
|  | 4 | 14.267 | 13.134 |
|  | 5 | 20.867 | 17.932 |
| LF/HF ratio | 1 | 5.984 | 6.862 |
|  | 2 | 4.266 | 4.408 |
|  | 3 | 2.795 | 1.742 |
|  | 4 | 5.263 | 4.497 |
|  | 5 | 4.547 | 4.697 |
| Salivary Copeptin (pmol·l⁻¹) | 1 | 2.307 | 0.726 |
|  | 2 | 2.079 | 0.428 |
|  | 3 | 2.504 | 0.679 |
|  | 4 | 2.334 | 0.588 |
|  | 5 | 2.360 | 0.681 |
