# Supplementary material for: Non-invasive biomarkers of perioperative stress in ophthalmic surgeons: Heart rate variability, cortisol and salivary copeptin in a feasibility study
Source: PLoS One. 2026 Jul 23;21(7):e0354245. doi: 10.1371/journal.pone.0354245 (PMC13395310; doi:10.1371/journal.pone.0354245)
Supplement: Peer review history

# Peer review: Decision Letter 0

**DOI:** 10.1371/journal.pone.0354245.r001

**Authors:** Tommaso Lomonaco

23 Apr 2026

PONE-D-25-67118

Non-invasive biomarkers of perioperative stress in ophthalmic surgeons: heart rate variability, cortisol and salivary copeptin in a feasibility study

PLOS One

Dear Dr. Adolfo Aracil-Marco,

Thank you for submitting your manuscript to PLOS ONE. After careful consideration, we feel that it has merit but does not fully meet PLOS ONE’s publication criteria as it currently stands. Therefore, we invite you to submit a revised version of the manuscript that addresses the points raised during the review process.

Please submit your revised manuscript by Jun 07 2026 11:59PM. If you will need more time than this to complete your revisions, please reply to this message or contact the journal office at plosone@plos.org. When you're ready to submit your revision, log on to https://www.editorialmanager.com/pone/ and select the 'Submissions Needing Revision' folder to locate your manuscript file.

Please include the following items when submitting your revised manuscript:

- A letter that responds to each point raised by the academic editor and reviewer(s). You should upload this letter as a separate file labeled 'Response to Reviewers'.
- A marked-up copy of your manuscript that highlights changes made to the original version. You should upload this as a separate file labeled 'Revised Manuscript with Track Changes'.
- An unmarked version of your revised paper without tracked changes. You should upload this as a separate file labeled 'Manuscript'.

If you would like to make changes to your financial disclosure, please include your updated statement in your cover letter. Guidelines for resubmitting your figure files are available below the reviewer comments at the end of this letter.

If applicable, we recommend that you deposit your laboratory protocols in protocols.io to enhance the reproducibility of your results. Protocols.io assigns your protocol its own identifier (DOI) so that it can be cited independently in the future. For instructions see: https://journals.plos.org/plosone/s/submission-guidelines#loc-laboratory-protocols. Additionally, PLOS ONE offers an option for publishing peer-reviewed Lab Protocol articles, which describe protocols hosted on protocols.io. Read more information on sharing protocols at https://plos.org/protocols?utm_medium=editorial-email&utm_source=authorletters&utm_campaign=protocols.

As the corresponding author, your ORCID iD is verified in the submission system and will appear in the published article. PLOS supports the use of ORCID, and we encourage all coauthors to register for an ORCID iD and use it as well. Please encourage your coauthors to verify their ORCID iD within the submission system before final acceptance, as unverified ORCID iDs will not appear in the published article. *Only*  the individual author can complete the verification step; PLOS staff *cannot*  verify ORCID iDs on behalf of authors.

We look forward to receiving your revised manuscript.

Kind regards,

Tommaso Lomonaco, Ph.D

Academic Editor

PLOS One

Journal requirements:

When submitting your revision, we need you to address these additional requirements.

1. Please ensure that your manuscript meets PLOS ONE's style requirements, including those for file naming. The PLOS ONE style templates can be found at

https://journals.plos.org/plosone/s/file?id=wjVg/PLOSOne_formatting_sample_main_body.pdf and

https://journals.plos.org/plosone/s/file?id=ba62/PLOSOne_formatting_sample_title_authors_affiliations.pdf

2. In the online submission form you indicate that your data is not available for proprietary reasons and have provided a contact point for accessing this data. Please note that your current contact point is a co-author on this manuscript. According to our Data Policy, the contact point must not be an author on the manuscript and must be an institutional contact, ideally not an individual. Please revise your data statement to a non-author institutional point of contact, such as a data access or ethics committee, and send this to us via return email. Please also include contact information for the third party organization, and please include the full citation of where the data can be found.

If the reviewer comments include a recommendation to cite specific previously published works, please review and evaluate these publications to determine whether they are relevant and should be cited. There is no requirement to cite these works unless the editor has indicated otherwise.

Additional Editor Comments:

Dear Authors, according to the reviewers' comments I would like to suggest major revisions. Please make sure to respond to all comments and criticisms raised from the reviewers.

Best regards,

Tommaso Lomonaco

[Note: HTML markup is below. Please do not edit.]

Reviewers' comments:

Reviewer's Responses to Questions

**Comments to the Author**

1. Is the manuscript technically sound, and do the data support the conclusions?

The manuscript must describe a technically sound piece of scientific research with data that supports the conclusions. Experiments must have been conducted rigorously, with appropriate controls, replication, and sample sizes. The conclusions must be drawn appropriately based on the data presented.

Reviewer #1: No

Reviewer #2: Partly

**********

2. Has the statistical analysis been performed appropriately and rigorously?

Reviewer #1: Yes

Reviewer #2: Yes

**********

3. Have the authors made all data underlying the findings in their manuscript fully available?

The PLOS Data policy requires authors to make all data underlying the findings described in their manuscript fully available without restriction, with rare exception (please refer to the Data Availability Statement in the manuscript PDF file). The data should be provided as part of the manuscript or its supporting information, or deposited to a public repository. For example, in addition to summary statistics, the data points behind means, medians and variance measures should be available. If there are restrictions on publicly sharing data—e.g. participant privacy or use of data from a third party—those must be specified.

Reviewer #1: Yes

Reviewer #2: Yes

**********

4. Is the manuscript presented in an intelligible fashion and written in standard English?

PLOS ONE does not copyedit accepted manuscripts, so the language in submitted articles must be clear, correct, and unambiguous. Any typographical or grammatical errors should be corrected at revision, so please note any specific errors here.

Reviewer #1: Yes

Reviewer #2: Yes

**********

5. Review Comments to the Author

Please use the space provided to explain your answers to the questions above. You may also include additional comments for the author, including concerns about dual publication, research ethics, or publication ethics. (Please upload your review as an attachment if it exceeds 20,000 characters)

Reviewer #1: The present study investigates salivary cortisol, copeptin, and heart rate variability (HRV) in ophthalmologists (n = 15) in relation to the timing of cataract surgery. Measurements were obtained on a rest day (single sampling) and on the surgery day (four time points in relation to the surgical procedure). The authors report a decrease in cortisol levels, an increase in copeptin levels, and several statistical changes in HRV parameters. Based on these findings, they conclude that copeptin might be a potential stress marker for evaluating the effects of stress on the body.

During the evaluation of the manuscript, several major issues were identified that should be addressed:

Participant selection

Why were only ophthalmologists included in this experiment? Considering that the number of participants is relatively small, including surgeons from similar surgical specialties could help increase the sample size and strengthen the study. Otherwise, the authors should clearly justify why ophthalmologists were specifically chosen.

Interpretation of copeptin results

Another important concern relates to the authors’ claim that copeptin levels increase in relation to surgery. However, based on the results presented, this interpretation does not appear to be supported by the data. Although there is a slight increase in copeptin levels during surgery, this change is not statistically significant. Without a statistically significant difference, it is not possible to suggest copeptin as a novel stress marker based on the current findings. Therefore, the conclusions presented in the abstract, as well as in the main text and discussion, appear to be overstated. The authors refer to a “tendency,” yet no corresponding p-value is reported.

Use of rest-day samples

The authors collected samples on the preceding rest day. However, these values were not used for statistical comparisons in the results presented in the text, tables, or figures. If the resting values were not used for comparison with the surgery-day values, it is unclear why they were collected. In principle, surgery-day measurements should be compared with resting values. If the authors have an alternative rationale, it should be clearly explained.

Correlation analyses

It is unclear why correlation analyses were performed separately for the rest day and the surgery day. The authors should provide a justification for this analytical approach.

Interpretation of copeptin findings and Figure 3c

The most novel aspect of the manuscript is the measurement of copeptin levels in saliva. However, the conclusions drawn are not statistically supported by the data and do not adequately substantiate the authors’ claims. Furthermore, in Figure 3c, the cortisol/copeptin ratio is presented. Since copeptin levels did not show a significant change, this figure essentially represents an exaggerated version of Figure 3a (cortisol levels). In effect, the ratio reflects the division of cortisol values by relatively unchanged copeptin values, which may artificially amplify the apparent pattern observed for cortisol.

Reviewer #2: Non-invasive biomarkers of perioperative stress in ophthalmic surgeons: heart rate variability, cortisol and salivary copeptin in a feasibility study

Dear author,

Thank you for your time and dedication in conducting this interesting work. The author has conducted the study with a clear orientation. However, some weak points need to be addressed before it’s suitable for publication in the journal. Minor revision is recommended, and comments are appended below.

Abstract

If it is within the word limit specified by the journal guidelines, including the mean, SD, and p-value for statistical significance (for example, the notation in lines 46-47) may convince the reader of the study’s integrity and transparency.

The summary should be based on the objective and the result of the study. For instance, the stress levels increase/decrease…… and then suggest the application of HRV and hormonal change as biomarkers of perioperative stress.

Keyword

Surgeon, physician, or other words, to reflect the study’s subject, should be included in this section. HR can be removed if including those words exceeds the limited keywords.

Introduction

Lines 71-72, SDNN should be the standard deviation of normal-to-normal RR intervals.

Line 75, including more references http such as://doi.org/10.3389/fpubh.2017.00258.

Line 80-84, It would be clearer to reorganise this section by introducing the copeptin and explaining why it is of interest, followed by the origin of this hormone, and concluding with its significance in the medical field.

Materials and methods

The data recording process is unclear. The author indicated that the HR sensor and strap were fixed on the subject’s chest, right? This suggests that data could be recorded continuously throughout the study (pre-surgery, surgery, and post-surgery), with the interval for HRV data analysis scheduled according to preferences. Why did the author choose to record the ultra-short-term HRV (<5 min) in this study? What is the time interval of data recording? I am familiar with the Kubios HRV scientific software program, which allows the researcher to select the time interval for data analysis to fit the study objective. Please clarify or discuss in more detail in the section.

Results

The author should report the results (mean SD) of the measured HRV metric rather than just only the correlation between parameters to persuade the reader of the study’s transparency.

Based on the above comment, the correlation between HRV metrics, such as RMSSD and pNN50, the indicator reflecting vagal dominance, is predictable and has been reported elsewhere. That’s why the mean SD values of the HRV metric and other parameters, along with a significant difference, should be reported as mentioned above.

Lines 198-199: “However, correlational analyses performed within each measurement didn’t replicate this finding” This sentence is unclear. Please clarify or revise it accordingly.

Discussion

The summary does not align with the abstract. Please revise them to ensure consistency.

Additional comments

The notion of figure should be placed at the end of the sentence. For example, in line 88

**********

6. PLOS authors have the option to publish the peer review history of their article (what does this mean?). If published, this will include your full peer review and any attached files.

If you choose “no”, your identity will remain anonymous but your review may still be made public.

**Do you want your identity to be public for this peer review?**  For information about this choice, including consent withdrawal, please see our Privacy Policy.

Reviewer #1: No

Reviewer #2: **Yes:** Metha Chanda

**********

[NOTE: If reviewer comments were submitted as an attachment file, they will be attached to this email and accessible via the submission site. Please log into your account, locate the manuscript record, and check for the action link "View Attachments". If this link does not appear, there are no attachment files.]

To ensure your figures meet our technical requirements, please review our figure guidelines: https://journals.plos.org/plosone/s/figures

You may also use PLOS’s free figure tool, NAAS, to help you prepare publication quality figures: https://journals.plos.org/plosone/s/figures#loc-tools-for-figure-preparation.

NAAS will assess whether your figures meet our technical requirements by comparing each figure against our figure specifications.

# Author comment: Author response to Decision Letter 1

**DOI:** 10.1371/journal.pone.0354245.r002

28 May 2026

Response to reviewers:

Reviewer #1:

The present study investigates salivary cortisol, copeptin, and heart rate variability (HRV) in ophthalmologists (n = 15) in relation to the timing of cataract surgery. Measurements were obtained on a rest day (single sampling) and on the surgery day (four time points in relation to the surgical procedure). The authors report a decrease in cortisol levels, an increase in copeptin levels, and several statistical changes in HRV parameters. Based on these findings, they conclude that copeptin might be a potential stress marker for evaluating the effects of stress on the body.

During the evaluation of the manuscript, several major issues were identified that should be addressed:

Participant selection

Why were only ophthalmologists included in this experiment? Considering that the number of participants is relatively small, including surgeons from similar surgical specialties could help increase the sample size and strengthen the study. Otherwise, the authors should clearly justify why ophthalmologists were specifically chosen.

R: We thank the reviewer for this constructive comment. The decision to include exclusively ophthalmologists was intentional and based on the need for a homogeneous surgical model. Ophthalmic surgery is characterized by high-precision microsurgery performed under a microscope, which involves a specific type of cognitive load and fine motor stress that differs significantly from larger-scale surgical specialties. Including other specialties would have introduced significant confounding variables related to different surgical ergonomics and duration. However, we agree with the reviewer’s point on sample size; we have now expanded the Participants subheading at the Methods section to explicitly justify the selection of this specialty and have acknowledged the small sample size as a limitation, emphasizing that this study serves as a specialized model for high-precision surgical stress.

Interpretation of copeptin results

Another important concern relates to the authors’ claim that copeptin levels increase in relation to surgery. However, based on the results presented, this interpretation does not appear to be supported by the data. Although there is a slight increase in copeptin levels during surgery, this change is not statistically significant. Without a statistically significant difference, it is not possible to suggest copeptin as a novel stress marker based on the current findings. Therefore, the conclusions presented in the abstract, as well as in the main text and discussion, appear to be overstated. The authors refer to a “tendency,” yet no corresponding p-value is reported.

R: We sincerely thank the reviewer for this rigorous observation. We acknowledge that the increase in copeptin levels did not reach the threshold of statistical significance (p < .05) and that describing this as a definitive 'increase' or 'marker' was an overstatement in the original draft. We have thoroughly revised the manuscript to reflect this. We now report the exact p-values and have replaced definitive claims with more cautious terminology, such as 'non-significant trend' or 'preliminary observation.' Furthermore, we have adjusted the conclusions to emphasize that while copeptin shows potential, our current data do not support its use as a definitive stress marker in this specific context without further large-scale validation.

Use of rest-day samples

The authors collected samples on the preceding rest day. However, these values were not used for statistical comparisons in the results presented in the text, tables, or figures. If the resting values were not used for comparison with the surgery-day values, it is unclear why they were collected. In principle, surgery-day measurements should be compared with resting values. If the authors have an alternative rationale, it should be clearly explained.

R: We appreciate the reviewer’s comment and apologize for any lack of clarity regarding the role of the rest day measurements. These samples were collected on a separate day (not the day immediately preceding surgery) to serve as an independent baseline reference for the participants' physiological status at the same time of day (8:00 AM) without the influence of surgical anticipation. We considered these as independent reference values rather than a factor within the repeated measures ANOVA, which was specifically designed to evaluate the acute fluctuations during the surgical day. To address the reviewer’s concern, we have explicitly compared the rest-day baseline with the surgery-day baseline using a Student’s paired t-test (as detailed in the Methods and Results) and have clarified in the text that this comparison serves to establish whether participants started the surgical day at a significantly different physiological state than on a typical rest day.

Correlation analyses

It is unclear why correlation analyses were performed separately for the rest day and the surgery day. The authors should provide a justification for this analytical approach.

R: As stated above, we considered that both days at which the measurements were taken were independent. Therefore, we performed the correlational analyses within each particular day to explore for potential divergent associations induced by the subjects exposure to surgical stress.

Interpretation of copeptin findings and Figure 3c

The most novel aspect of the manuscript is the measurement of copeptin levels in saliva. However, the conclusions drawn are not statistically supported by the data and do not adequately substantiate the authors’ claims. Furthermore, in Figure 3c, the cortisol/copeptin ratio is presented. Since copeptin levels did not show a significant change, this figure essentially represents an exaggerated version of Figure 3a (cortisol levels). In effect, the ratio reflects the division of cortisol values by relatively unchanged copeptin values, which may artificially amplify the apparent pattern observed for cortisol.

R: We appreciate the reviewer’s critical assessment of the salivary copeptin data and the cortisol/copeptin ratio. Our original intention in including Figure 3c was to explore the divergent temporal dynamics between these two hormones during surgical stress. However, we take the reviewer's point that since the fluctuations in copeptin levels did not reach statistical significance, presenting this ratio could potentially overemphasize or artificially amplify the patterns observed in cortisol levels. To maintain the highest level of statistical transparency and avoid any overstatement of our findings, we have removed Figure 3c and all associated discussion regarding this ratio from the revised manuscript. We believe this adjustment ensures that our conclusions are more strictly aligned with the significant data presented.

Reviewer #2:

Non-invasive biomarkers of perioperative stress in ophthalmic surgeons: heart rate variability, cortisol and salivary copeptin in a feasibility study

Dear author,

Thank you for your time and dedication in conducting this interesting work. The author has conducted the study with a clear orientation. However, some weak points need to be addressed before it’s suitable for publication in the journal. Minor revision is recommended, and comments are appended below.

Abstract

If it is within the word limit specified by the journal guidelines, including the mean, SD, and p-value for statistical significance (for example, the notation in lines 46-47) may convince the reader of the study’s integrity and transparency.

R: We fully agree with the reviewer that transparency in reporting statistical data is essential for scientific integrity. Following this recommendation, we have revised the Results section and the Abstract to include the mean, standard deviation (SD), and exact p-values for all primary and secondary outcomes along the manuscript -not necessarily at the abstract-, regardless of whether they reached statistical significance. This ensures that the reader has full access to the magnitude of the effects and the precision of our estimates.

The summary should be based on the objective and the result of the study. For instance, the stress levels increase/decrease…… and then suggest the application of HRV and hormonal change as biomarkers of perioperative stress.

R: We appreciate the reviewer’s suggestion to refine the summary. However, we would like to clarify that our study did not measure 'perceived stress' through psychometric scales; instead, it focused on the objective physiological response to a standardized occupational stressor (routine cataract surgery). Therefore, we cannot report whether the participants 'felt' more or less stressed. To avoid ambiguity, we have revised the summary and conclusions to focus strictly on the 'biomarker response' and 'neuroendocrine activation' rather than 'stress levels.'

Keyword

Surgeon, physician, or other words, to reflect the study’s subject, should be included in this section. HR can be removed if including those words exceeds the limited keywords.

R: We thank the reviewer for this practical suggestion to improve the visibility of our study. We agree that including terms related to the study population will enhance its discoverability in academic databases. Accordingly, we have updated the keywords section to include 'Surgeon' and 'Physician.' To comply with the journal's constraints on the number of keywords, we have removed 'HR' (Heart Rate), as the physiological aspects are already represented by 'Heart Rate Variability' and other hormonal markers.

Introduction

Lines 71-72, SDNN should be the standard deviation of normal-to-normal RR intervals.

R: We are grateful to the reviewer for pointing out this technical detail. We have corrected the definition of SDNN in the 'Methods' section to ensure it strictly follows the international standards for heart rate variability (HRV) analysis. The text now correctly defines SDNN as the standard deviation of normal-to-normal (NN) intervals.

Line 75, including more references http such as://doi.org/10.3389/fpubh.2017.00258.

R: This reference has been added.

Line 80-84, It would be clearer to reorganise this section by introducing the copeptin and explaining why it is of interest, followed by the origin of this hormone, and concluding with its significance in the medical field.

R: While we had aimed to follow this logical flow in the original draft, we appreciate the reviewer’s suggestion to make this structure more explicit. We have revised the Introduction to more clearly follow the proposed sequence: (1) introducing copeptin and its interest as a stable surrogate for vasopressin, (2) explaining its physiological origin and biochemical properties, and (3) detailing its clinical significance as a biomarker of acute stress in the medical and surgical fields. We believe this reorganization significantly improves the clarity and readability of the theoretical framework.

Materials and methods

The data recording process is unclear. The author indicated that the HR sensor and strap were fixed on the subject’s chest, right? This suggests that data could be recorded continuously throughout the study (pre-surgery, surgery, and post-surgery), with the interval for HRV data analysis scheduled according to preferences. Why did the author choose to record the ultra-short-term HRV (<5 min) in this study? What is the time interval of data recording? I am familiar with the Kubios HRV scientific software program, which allows the researcher to select the time interval for data analysis to fit the study objective. Please clarify or discuss in more detail in the section.

R: We sincerely thank the reviewer for this insightful comment. We would like to clarify the rationale behind our recording protocol. While the HR sensor and strap were indeed used, they were not kept on the surgeons throughout the entire surgical day to ensure maximum comfort and avoid any interference with surgical ergonomics and sterile conditions. Consequently, we opted for ultra-short-term (2-minute) recordings at specific key moments, as this approach was the least invasive for the natural course of the procedure. R: We recognize the capabilities of software like Kubios for analyzing continuous data; however, our priority was to ensure the surgeon's performance was not compromised by the equipment. Following your suggestion, we have clarified the time intervals in the 'Methods' section and added a dedicated paragraph in the 'Discussion' regarding the use and limitations of ultra-short-term HRV in clinical settings.

Results

The author should report the results (mean SD) of the measured HRV metric rather than just only the correlation between parameters to persuade the reader of the study’s transparency.

Based on the above comment, the correlation between HRV metrics, such as RMSSD and pNN50, the indicator reflecting vagal dominance, is predictable and has been reported elsewhere. That’s why the mean SD values of the HRV metric and other parameters, along with a significant difference, should be reported as mentioned above.

R: We appreciate the reviewer’s emphasis on transparency. Our primary objective was to avoid redundant data presentation, as the mean and standard deviation for the HRV metrics are already illustrated in the figures provided. We followed the principle of not duplicating information across text, tables, and figures to maintain conciseness. However, we understand the reviewer's preference for precise numerical values to facilitate future meta-analyses or detailed comparisons. Therefore, we have now included a new comprehensive table [Supplementary Table S1] containing the exact mean and SD for all HRV parameters across all time points. We have also added the specific RMSSD and pNN50 values in the text where correlations are discussed to provide immediate context. We believe this addition addresses the need for transparency while preserving the visual impact of our figures.

Lines 198-199: “However, correlational analyses performed within each measurement didn’t replicate this finding” This sentence is unclear. Please clarify or revise it accordingly.

R: We are grateful to the reviewer for highlighting the lack of clarity in this sentence. Upon internal review, we realized that the statement was indeed ambiguous and did not add significant value to the interpretation of the results. To ensure a more cohesive and direct narrative, we have removed this sentence from the revised manuscript. We believe this adjustment improves the overall flow and clarity of the Results section.

Discussion

The summary does not align with the abstract. Please revise them to ensure consistency.

R: We thank the reviewer for identifying this inconsistency. We have thoroughly reviewed and revised both the Abstract and the Summary/Conclusion sections to ensure they are perfectly aligned. Specifically, we have updated both sections to reflect the lack of statistical significance in copeptin fluctuations, focusing instead on the significant changes observed in HRV and cortisol. We have also ensured that the terminology and the strength of our conclusions are consistent throughout the entire manuscript, avoiding any overstatements that were previously present in the Abstract.

Additional comments

The notion of figure should be placed at the end of the sentence. For example, in line 88

R: We appreciate the reviewer's attention to detail regarding the manuscript's formatting. Although we did not find a figure reference in line 88, we assume the reviewer may have been referring to line 188 or similar instances. Following this suggestion, we have carefully reviewed the entire manuscript and ensured that all figure citations are now placed at the end of the corresponding sentences (e.g., '...as observed in the results (Figure 1).'). This formatting has been applied consistently throughout the revised text

Attachment Submitted filename: PONE -D-25-67118-Response to reviewers.docx

# Peer review: Decision Letter 1

**DOI:** 10.1371/journal.pone.0354245.r003

**Authors:** Tommaso Lomonaco

6 Jul 2026

<p>Non-invasive biomarkers of perioperative stress in ophthalmic surgeons: heart rate variability, cortisol and salivary copeptin in a feasibility study

PONE-D-25-67118R1

Dear Dr. Adolfo Aracil-Marco,

We’re pleased to inform you that your manuscript has been judged scientifically suitable for publication and will be formally accepted for publication once it meets all outstanding technical requirements.

Within one week, you’ll receive an e-mail detailing the required amendments. When these have been addressed, you’ll receive a formal acceptance letter and your manuscript will be scheduled for publication.

An invoice will be generated when your article is formally accepted. Please note, if your institution has a publishing partnership with PLOS and your article meets the relevant criteria, all or part of your publication costs will be covered. Please make sure your user information is up-to-date by logging into Editorial Manager at Editorial Manager® and clicking the ‘Update My Information' link at the top of the page. For questions related to billing, please contact billing support.

If your institution or institutions have a press office, please notify them about your upcoming paper to help maximize its impact. If they’ll be preparing press materials, please inform our press team as soon as possible -- no later than 48 hours after receiving the formal acceptance. Your manuscript will remain under strict press embargo until 2 pm Eastern Time on the date of publication. For more information, please contact onepress@plos.org.

Kind regards,

Tommaso Lomonaco, Ph.D

Academic Editor

PLOS One

Reviewers' comments:

Reviewer's Responses to Questions

**Comments to the Author**

1. If the authors have adequately addressed your comments raised in a previous round of review and you feel that this manuscript is now acceptable for publication, you may indicate that here to bypass the “Comments to the Author” section, enter your conflict of interest statement in the “Confidential to Editor” section, and submit your "Accept" recommendation.

Reviewer #1: All comments have been addressed

Reviewer #2: All comments have been addressed

**********

2. Is the manuscript technically sound, and do the data support the conclusions?

The manuscript must describe a technically sound piece of scientific research with data that supports the conclusions. Experiments must have been conducted rigorously, with appropriate controls, replication, and sample sizes. The conclusions must be drawn appropriately based on the data presented.

Reviewer #1: Yes

Reviewer #2: Yes

**********

3. Has the statistical analysis been performed appropriately and rigorously?

Reviewer #1: Yes

Reviewer #2: Yes

**********

4. Have the authors made all data underlying the findings in their manuscript fully available?

The PLOS Data policy requires authors to make all data underlying the findings described in their manuscript fully available without restriction, with rare exception (please refer to the Data Availability Statement in the manuscript PDF file). The data should be provided as part of the manuscript or its supporting information, or deposited to a public repository. For example, in addition to summary statistics, the data points behind means, medians and variance measures should be available. If there are restrictions on publicly sharing data—e.g. participant privacy or use of data from a third party—those must be specified.

Reviewer #1: Yes

Reviewer #2: Yes

**********

5. Is the manuscript presented in an intelligible fashion and written in standard English?

PLOS ONE does not copyedit accepted manuscripts, so the language in submitted articles must be clear, correct, and unambiguous. Any typographical or grammatical errors should be corrected at revision, so please note any specific errors here.

Reviewer #1: Yes

Reviewer #2: Yes

**********

6. Review Comments to the Author

Please use the space provided to explain your answers to the questions above. You may also include additional comments for the author, including concerns about dual publication, research ethics, or publication ethics. (Please upload your review as an attachment if it exceeds 20,000 characters)

Reviewer #1: Please shorten the title (for example there is no need to say "a feasibility study)

Correlation graphs are not very much necessary, they might be presented as text. The first lines of the correlation tables are unnecessary (correlation between the same parameters, i.e. empty cells)

Reviewer #2: Dear authors,

Your responses are musch satisfied and could improve the study's integrity and transparency. I believe that the manuscript is suitable for publication in the journal in its current form.

Best regards,

Reviewer

**********

7. PLOS authors have the option to publish the peer review history of their article (what does this mean?). If published, this will include your full peer review and any attached files.

If you choose “no”, your identity will remain anonymous but your review may still be made public.

**Do you want your identity to be public for this peer review?**  For information about this choice, including consent withdrawal, please see our Privacy Policy.

Reviewer #1: **Yes:** SEDAT YILDIZ

Reviewer #2: **Yes:** Metha Chanda

**********

# Editor report: Acceptance letter

**DOI:** 10.1371/journal.pone.0354245.r004

**Authors:** Tommaso Lomonaco

PONE-D-25-67118R1

PLOS One

Dear Dr. Aracil-Marco,

I'm pleased to inform you that your manuscript has been deemed suitable for publication in PLOS One. Congratulations! Your manuscript is now being handed over to our production team.

At this stage, our production department will prepare your paper for publication. This includes ensuring the following:

* All references, tables, and figures are properly cited

* All relevant supporting information is included in the manuscript submission,

* There are no issues that prevent the paper from being properly typeset

You will receive further instructions from the production team, including instructions on how to review your proof when it is ready. Please keep in mind that we are working through a large volume of accepted articles, so please give us a few days to review your paper and let you know the next and final steps.

Lastly, if your institution or institutions have a press office, please let them know about your upcoming paper now to help maximize its impact. If they'll be preparing press materials, please inform our press team within the next 48 hours. Your manuscript will remain under strict press embargo until 2 pm Eastern Time on the date of publication. For more information, please contact onepress@plos.org.

You will receive an invoice from PLOS for your publication fee after your manuscript has reached the completed accept phase. If you receive an email requesting payment before acceptance or for any other service, this may be a phishing scheme. Learn how to identify phishing emails and protect your accounts at https://explore.plos.org/phishing.

If we can help with anything else, please email us at customercare@plos.org.

Thank you for submitting your work to PLOS ONE and supporting open access.

Kind regards,

PLOS ONE Editorial Office Staff

on behalf of

Prof. Tommaso Lomonaco

Academic Editor

PLOS One
